# Incorporating VR-RENDER Fusion Software in Robot-Assisted Partial Prostatectomy: The First Case Report

**DOI:** 10.3390/curroncol30020131

**Published:** 2023-01-31

**Authors:** Che-Hsueh Yang, Li-Hsun Chen, Yi-Sheng Lin, Chao-Yu Hsu, Min-Che Tung, Shih-Wei Huang, Chi-Hsiang Wu, Yen-Chuan Ou

**Affiliations:** 1 Division of Urology, Department of Surgery, Tungs’ Taichung MetroHarbor Hospital, Taichung 435, Taiwan; 2 Asian Institute of Telesurgery, Chang Bing Show Chwan Memorial Hospital, Changhua 505, Taiwan; 3 Division of General Surgery, Department of Surgery, Chang Bing Show Chwan Memorial Hospital, Changhua 505, Taiwan; 4 Department of Urology, Chang Bing Show Chwan Memorial Hospital, Changhua 505, Taiwan

**Keywords:** robotic surgical procedures/adverse effects, prostatic neoplasms/pathology, prostatic neoplasms/diagnostic imaging, treatment outcome, feasibility studies, case reports

## Abstract

Currently, the active surveillance of men with favorable intermediate-risk localized prostate cancer (PCa) is a longstanding controversy, in terms of their oncological outcomes, and radical prostatectomy would constitute a similar concern of overtreatment, regarding its functional outcomes. Thus, focal therapy could be considered in men belonging to favorable intermediate-risk group. Among all focal therapies, high-intensity focused ultrasound (HIFU) was the most studied methodology in clinical trials. Although HIFU provided better functional outcomes than radical prostatecomy, the oncological outcomes were inferior in men with intermediate-risk localized PCa. Two articles have been published discussing the feasibility and clinical outcomes of robot-assisted partial prostatectomy (RAPP), and both the functional and oncological outcomes were superior than those with HIFU. However, the rate of positive surgical margins (PSMs) was reported as high in the literature. Here, we present a case of favorable intermediate-risk localized PCa with an isolated tumor at the anterior apex. After reconstructing a personal three-dimensional (3D) image, we utilized it in a 3D image-guided precise excise, followed by intraoperative frozen specimen review. We found that this method may present a resolution to the high PSM rate documented in the current literature regarding RAPP. This method merits further study with a well-designed prospective study.

## 1. Introduction

According to the National Comprehensive Cancer Network (NCCN) guidelines, men with localized prostate cancer (PCa) can be categorized into six subgroups: very low risk, low risk, favorable intermediate risk, unfavorable intermediate risk, high risk, and very high risk [1,2]. In men with very low/low risk, active surveillance (AS) is suggested to them when life expectancy is longer than 10 years. In a study of large cohorts over a 10- or 15-year period, distant metastasis or cancer-related death under the AS strategy in clinically insignificant localized PCa were quite rare—less than 1% [3,4]. However, when AS strategy is extended to the favorable intermediate-risk, careful consideration should be kept in mind when selecting suitable patients, and the tailoring monitoring strategies, such as prostate specific antigen (PSA) > 15 ng/mL or Gleason Grade group 2 [5], regarding when to repeat the biopsies, or re-assess their conditions are paramount [6]. Otherwise, the outcomes of cancer-specific/overall survival in men under AS would be inferior to those under active treatment [7]. As for the men with unfavorable intermediate-risk localized PCa and life expectancy over 10 years, radical prostatectomy (RP) is the treatment of choice [2].

Robot-assisted RP (RARP) could primarily benefit men with oncological outcomes such as lower chances of positive surgical margins (PSM), lower biochemical recurrent rate, and longer cancer-specific survival [8,9]. Moreover, significantly fewer men with RARP would feel regret after operation [10]. Although meta-analysis of randomized controlled trials demonstrated the beneficial effects of RARP on functional outcomes, such as erectile function and continence [9], a large-scale, non-randomized multicentral trial found that there was still a 27% incontinence rate and a 66% erectile dysfunction (ED) rate at 8 years after RARP [8]. These functional outcomes were usually recorded with patient-reported data and could vary significantly with the selection of a different baseline [11]. However, maximizing functional outcomes remains one of the primary considerations when treating localized PCa.

As mentioned above, AS was effective for men with very low-/low-risk localized PCa, as was RP for men with unfavorable intermediate-risk localized PCa. However, AS should be adopted carefully in certain scenarios of favorable intermediate-risk localized PCa, and RP may compromise functional outcomes after operation. Recently, the popularity of focal therapies is increasing greatly. Their main advantage lies in their ability to simultaneously preserve functional outcomes and control oncological outcomes. The best-known technique is high-intensity focused ultrasound (HIFU). In the past 5 years, there have been 27 HIFU clinical trials reported [12]. However, large-scale reports on the subject of partial prostatectomy (PP) were few [13,14,15]. Although precise excision was theoretically important in PP for oncological outcomes, no specific methods of localization were mentioned in these studies. With the advances of 3T multi-parametric magnetic resonance imaging (mpMRI) and of Prostate Imaging Reporting and Data System (PI-RADS), localized PCa could be more easily identified and biopsied under targeted methods. At the same time, remnant PCa could be excluded with the greatest possible accuracy. In animal models, incorporating enhanced reality images with VR-RENDER Fusion software would help identify the major anatomical planes, including vascular boundaries and target lesions [16]. As a result, we proposed a new image-guided method incorporating a reconstructed three-dimensional image into the surgeon’s console of the da Vinci Surgical System (Intuitive Surgical, Inc., Sunnyvale, CA, USA), and performed the precise excision of localized PCa at the anterior lobe after MRI-ultrasound fusion biopsy.

## 2. Case

### 2.1. Initial Assessments

A 62-year-old man’s PSA was measured every six months for two years, and the data had fluctuated to a wide range. The past records of his PSA, before coming to our clinics, were 5.178 ng/mL in October 2019, 5.442 ng/mL in April 2020, 4.343 ng/mL in October 2020, 3.520 ng/mL in April 2021, 3.892 ng/mL in October 2021, 5.243 ng/mL in April 2021, and 5.536 ng/mL in October 2021. All the PSA investigations were accompanied by digital rectal exams (DRE), and no suspicious hard nodules were palpated. In October 2021, he underwent a biopsy performed by the transrectal ultrasound (TRUS)-guided method, and benign hyperplasia was found in all 12 systemic cores.

**Figure 1:**
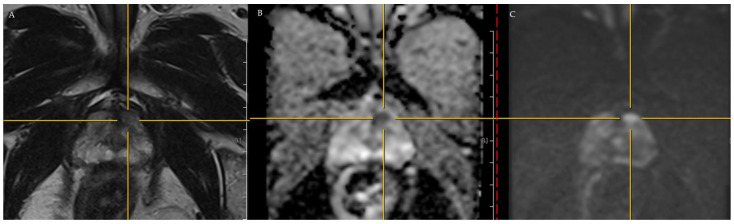
3T mpMRI assessment of the prostate. There was a PI-RADS version 2 score 4 lesion at the left anterior peripheral zone. (**A**) T2-weighted image. (**B**) Apparent diffusion coefficient. (**C**) Diffusion-weighted imaging.

**Figure 2:**
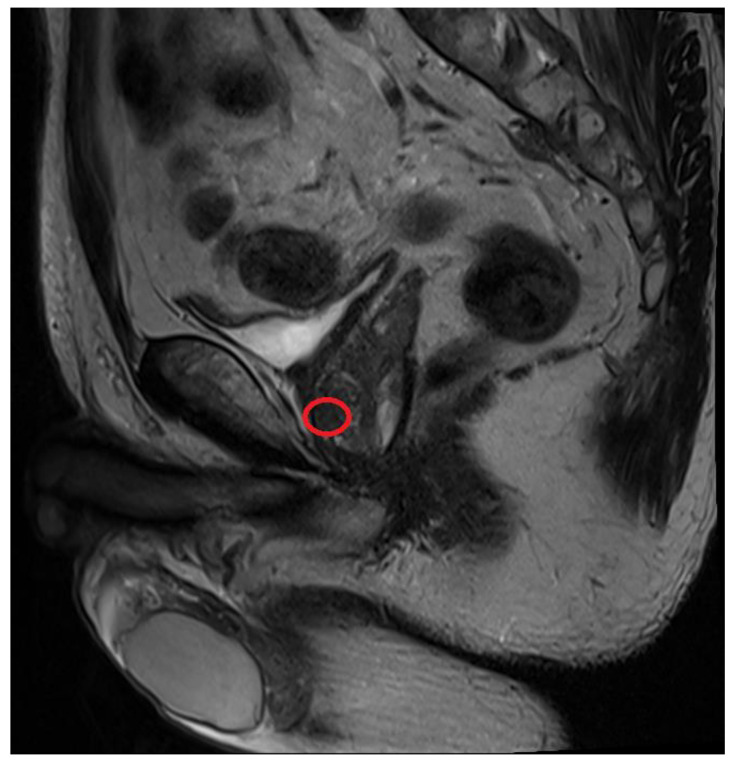
Suspicious lesion on the sagittal plane of T2-weighted image. The lesion was located at the apex of the prostate (red circle).

In February 2022, he came to our clinics, and the PSA in his blood sample showed 5.714 ng/mL. The prostate was still elastic, without a palpable hard nodule when performing DRE. After discussion, we recommended 3T mpMRI (Figure 1) to assess the general condition of his prostate and screen for any suspicious lesions. After completing 3T mpMRI, a suspicious lesion, measuring 0.4 × 0.2 cm, with markedly focal hypointense on apparent diffusion coefficient (ADC) and hyperintense on high b-value diffusion-weighted imaging (DWI) was found over the left-anterior peripheral zone of the prostate apex. This lesion was assessed as PI-RADS version 2 score 4 (Figure 1 and Figure 2).

After further discussion with the patient, we performed MRI/ultrasound prostate fusion biopsy on the suspicious lesion. A total of 6 cores of regions of interest (ROIs) and 12 cores of systemic biopsies were sampled. There was one core with Gleason 3+4 adenocarcinoma out of 6 cores of ROI and benign hyperplasia in all 12 cores of systemic biopsies. Based on the clinical information, he was diagnosed with favorable intermediate-risk localized PCa [2]. After explaining active surveillance and partial prostatectomy to the patient, he chose to participate in the robot-assisted PP (RAPP) project (IRB No. R111037, Tungs’ Taichung MetroHarbor Hospital, Taichung, Taiwan).

### 2.2. Creating the Personal 3D Model before RAPP

**Figure 3:**
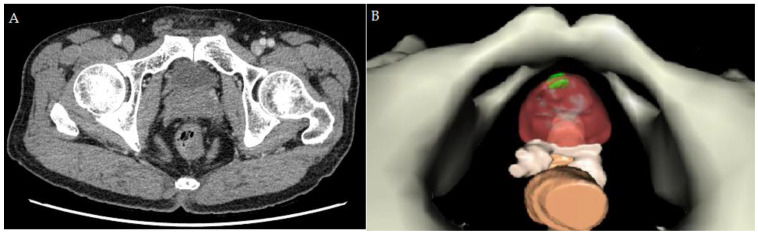
(**A**) The 1.25-mm thin slice for computed tomography used for reconstruction. (**B**) The reconstructed personal 3D image using for RAPP. The green area indicates the location of PCa.

To acquire better spatial resolution, an additional 1.25-mm thin slice of computed tomography (CT) images were used to create the 3D model. Multiplanar CT images were selected, and we applied color contrast to each layer and highlighted the anatomical details precisely. Then, we built the segmentation of the organs and vessels (Figure 3). The entire process was completed with VR-RENDER Fusion software (version 1.0, IRCAD, Strasbourg, France). Reconstructing the personal 3D model took 4 h.

### 2.3. RAPP

**Figure 4:**
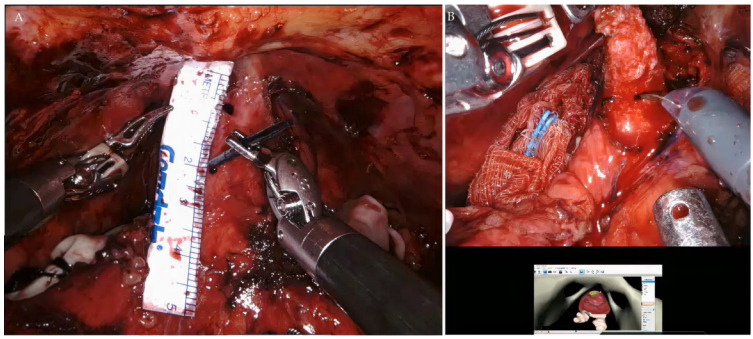
(**A**) Marking the area to be excised after exposing the prostate. (**B**) Demonstrating the reconstructed personal 3D image onto the console monitor while performing the RAPP.

The surgical steps were similar to our techniques of RARP [17,18,19,20]. After exposing the prostate, the reconstructed personal 3D image was introduced into the console of the da Vinci Surgical Xi System (Intuitive Surgical, Inc., Sunnyvale, CA, USA). We marked the area to be excised, and displayed the personal 3D image on the console monitor surface while performing RAPP (Figure 4).

**Figure 5:**
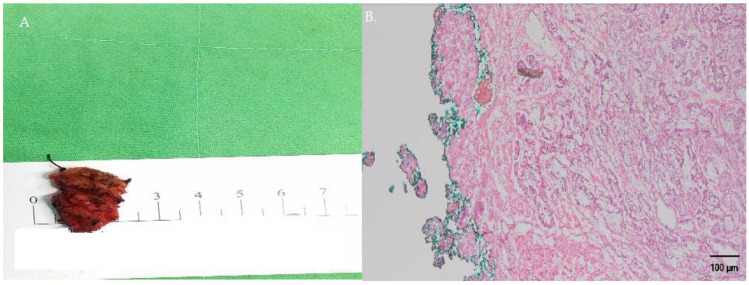
(**A**) The general excised specimen. After precise excision, the frozen specimen was sent to a single pathologist and reviewed immediately. (**B**) The intraoperative frozen review confirmed that there were PCa cells excised with negative surgical margins.

**Figure 6:**
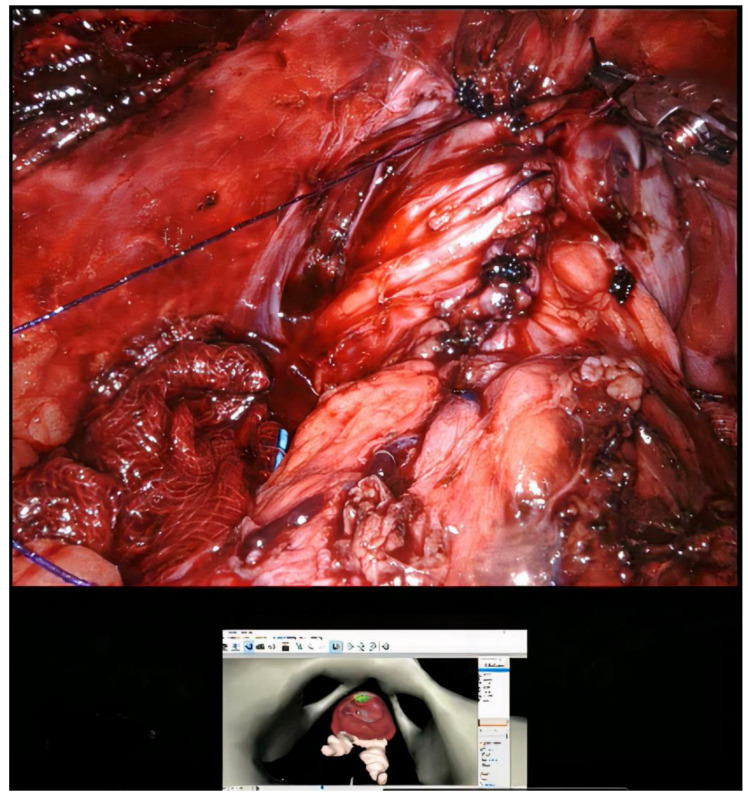
After intraoperative frozen review, the excised site was closed with watertight suture.

The precise excision was carried out, immediately followed by intraoperative frozen specimen review. The process of reviewing the frozen specimen took 27 min. After confirming there was PCa in the specimen and negative surgical margins (Figure 5), the excised site was sealed with a watertight suture with 3-0 V-Loc™ (Minneapolis, MN, USA) (Figure 6). The total console time was 96 min.

### 2.4. Oncological Outcome and Functional Outcome

**Figure 7:**
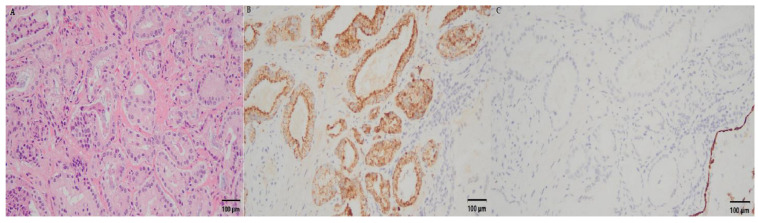
The Gleason score of the excised specimen was 3 + 4. (**A**) Microscopically, it revealed angulated and elongated acini that were haphazardly distributed in the stroma. (**B**) The tumor acini were positive for AMACR; these were negative in the basal layer. (**C**) The tumor was stained with p63 immunostains.

After the urethra catheter was removed and micturition was observed, the discharge was arranged. The total hospital stay was 7 days, and postoperative clinical appointments were arranged at every 3 months after discharge. The formal pathological report revealed that this PCa was evaluated as a Gleason score 3 + 4, and there was no evidence of metastasis to the bilateral obturator lymph nodes (total lymph nodes removed: 6) (Figure 7). In the first clinical visit, the patient’s continence (0 pad) and potency (international index of erectile function: 22) were totally recovered. The patient’s PSA level was measured as 2.230 ng/mL. In the second clinical visit, the PSA level further decreased to 1.909 ng/mL.

## 3. Discussion

Introducing PSA into screening for PCa has greatly altered the distribution of PCa [21]; an increasing trend has been observed in intermediate- and high-risk localized PCa [22]. These two groups of patients were mostly managed with RP or radiation therapies. For RARP, as we mentioned above, recent 8-year follow-up studies indicated that it would still result in 27% incontinence and 66% ED. These two functional parameters will significantly impact quality of life and recovery of mental health, particularly incontinence [23]. Thus, consideration of the issue of overtreatment is increasingly important [24]. With HIFU, the reported continence rate was 95.5% in follow-ups after a median time of 12 months; erectile function was decreased by 12% [12].

Although selecting focal ablation could significantly improve the recovery of continence and potency [25], the location of PCa at the anterior and apical position would make ablation difficult to carry out. As with the case in this study, isolated PCa at the anterior and apical position would be close to the sphincter and surrounded by the neurovascular bundle; ablation at this position would compromise the functional outcomes after treatment. Furthermore, the oncological outcomes would be less effective [26]. However, prostate hyperplasia underneath the anterior PCa would possibly act as a barrier preventing the tumor from extending posteriorly [27]; this position could be easily visualized, and the excision could be performed with precision. In this case, after exposing the prostate, the PCa could be identified under 3D image guidance. Intraoperative review of the frozen pathological specimens would enable us to confirm an effective excision with the detection of the presence of PCa, without PSM. In one study [13], researchers performed intraoperative systemic biopsies on the residual prostate to confirm an adequate excision. However, there were 11 PSM out of 88 cases of PP, and 4 (36%) of them were above 3 mm. In this study [13], 6 (6.8%) men underwent removal of the remnant prostate, and 2 out of these 6 men needed adjuvant radiation therapy for oncological control. Similarly, in the other study regarding PP, the rate of PSM was as high as 53%. In this regard, our 3D image-guided precise excision with intraoperative frozen specimen review might be the solution for lowering PSM rates.

In patients who have undergone RAPP, since both the residual prostatic tissues and cancerous cells would contribute to PSA alterations, the definition of biochemical failure should reasonably refer to that of the focal treatment. The optimal judgement of biochemical failure was the detection of the first nadir and the median nadir after RAPP; this was approximately 0.4 ng/mL [15]. PSA nadir + 1.0 ng/mL at 12 months and 1.5 ng/mL at 24 to 36 months would have 100% sensitivity and over 96% negative predictive value [28]. With this definition, approximately 2.8% of men would have biochemical failure after RAPP at a median of 25 months of postoperative observation. This rate is far superior to a 26% biochemical failure rate at 32 months after HIFU [29].

In functional outcomes, the current literature indicated that 100% of men could recover continence (0–1 pad) from 3 to 12 months post-operation. The median duration until continence was achieved was 1 month [13,15]. Regarding potency, 90.2% of men recover potency at 12 months after RAPP, and the median time to recovering potency was 4 months [13]. In this case, the patient could likely reach potency and continence at 3 months after RAPP.

Judging from the published literature and this case, the functional outcomes after RAPP are better than those after RARP. Thus, RAPP could benefit patients diagnosed with favorable intermediate-risk localized PCa with better functional outcomes and without worries of overtreatment, especially for men with isolated anterior PCa. However, regarding oncological outcomes, the precise definition of biochemical failure has not been established. In the published literature, with the strict definition of biochemical failure after RARP, 15 out of 25 men (60%) experienced PSA elevation due to benign prostate tissues [13]. Therefore, it was more suitable to adhere to PSA monitoring strategies as focal therapies [13,28]. Although PSM was not necessarily related to biochemical failure or salvage surgeries [13], this issue needs to be further studied with well-designed models.

In this case, we proposed a method of 3D image-guided precise excision and intraoperative frozen specimen review. Unlike any of the examples documented in the previous literature regarding RAPP [13,14,15], the VR-RENDER Fusion software was used in this case. It was able to provide 3D virtual images helping urologists perform delicate preoperative planning, since its diagnostic accuracy on lateralization and localization was significantly better than standard CT [30]. Furthermore, its advantages in accurate localization are superior to standard CT for the detection of small lesions [31]. Since men with low-risk and low-volume localized PCa would constitute the main group for whom RAPP would be indicated, the adoption of VR-RENDER Fusion software could make RAPP more meticulously performed in the future. Since precise excision with negative surgical margins could be carried out with the greatest degree of efficiency, patients could look forward to improved oncological outcomes—another potential benefit of this method. Our limitation was that this single case report was only an initial technical feasibility study. A sophisticated prospective cohort study should be carried out to validate our viewpoint.

## 4. Conclusions

3D image-guided RAPP with intraoperative frozen specimen review could not only offer quick functional outcome recovery, but also provide the possibility of better oncological outcomes. This method is merits further investigation with a well-designed prospective cohort study.

## Data Availability

Reports on the data supporting this case are included in the article.
